# Osteosynthesis with Arthroscopy of a Nonunited Tibial Anterior Cruciate Ligament Avulsion Fracture 30 Years after Injury

**DOI:** 10.1155/2023/4681973

**Published:** 2023-12-05

**Authors:** Jun Tanaka, Takuya Ohno, Hiroki Ishikawa, Shogo Fujita, Hiroki Okamura, Shigeo Yamakami, Yoshifumi Kudo

**Affiliations:** ^1^Department of Orthopedic Surgery, Nippon Koukan Hospital, 1-2-1 Koukandori, Kawasaki-ku, Kawasaki, Kanagawa, Japan; ^2^Department of Orthopedic Surgery, Showa University School of Medicine, 1-5-8 Hatanodai, Shinagawa-ku, Tokyo, Japan

## Abstract

There are a few reports on the treatment of nonunited tibial anterior cruciate ligament (ACL) avulsion fractures. To our knowledge, this is the first report of a case of nonunited tibial ACL avulsion fracture 30 years after injury. A 36-year-old woman injured her knee during a road traffic accident 30 years ago. Since then, she had persistent knee instability that was left untreated. She presented to our clinic because of knee pain she had been experiencing for a week. Radiography and computed tomography revealed tibial ACL avulsion fracture nonunions. Screw fixation with arthroscopy was performed, and bone fusion was obtained. The knee injury and osteoarthritis outcome score improved from 24 points preoperatively to 83 points postoperatively. The nonunion of intercondylar eminence fractures of the tibia, even up to 30 years after the initial injury, can be treated by osteosynthesis with arthroscopy.

## 1. Introduction

The tibial anterior cruciate ligament (ACL) avulsion fracture is a cartilage epiphyseal avulsion of the ACL attachment area on the anterior medial side of the tibial eminence. It is an uncommon injury that occurs in children and adolescents. In people of this age, the ACL is thought to be stronger than the bone. In adults, the ACL is more likely to rupture before the bone gets damaged. Its incidence is approximately 3 out of 100,000 people [1]. Furthermore, there are only a few reports in the literature on nonunited tibial ACL avulsion fractures [2].

Tibial ACL avulsion fractures require appropriate treatment because of complications such as limited extension, instability, and residual pain, as well as nonunion and deformity, if not treated appropriately [3]. Previously reported treatment options for tibial ACL avulsion fractures include osteosynthesis by open reduction, osteosynthesis with arthroscopy, debridement with arthroscopy, and ACL reconstruction [4–7]. On the other hand, the treatment of tibial ACL avulsion fracture nonunions is even rarer and less frequently reported, and the optimal treatment modality has not been well established.

To the best of our knowledge, there has been no report of the treatment of a nonunited tibial ACL avulsion fracture with osteosynthesis 30 years after the initial injury. This is the first report of a nonunited tibial ACL avulsion fracture that was treated 30 years after the initial injury via screw fixation with arthroscopy with good bony fusion.

## 2. Case Presentation

A 36-year-old woman who injured her left knee during a road traffic accident 30 years ago was diagnosed with a simple bruise and left untreated. However, the injured knee remained unstable thereafter. In addition, one week before her first visit, she began experiencing knee pain when standing up.

Her physical examination revealed pain during flexion but no tenderness. The range of motion (ROM) of her left knee ranged between 0 and 140 degrees. Slight instability was observed in the anterior drawer test. The posterior drawer test, valgus stress test, varus stress test, and McMurray test were all negative.

**Figure 1:**
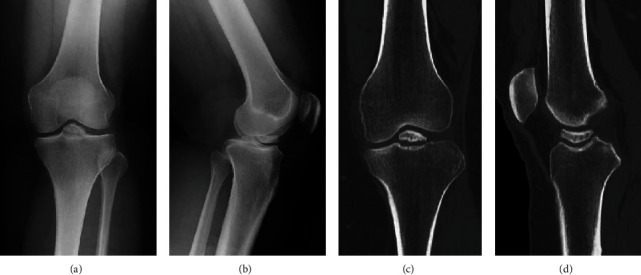
(a) Frontal view and (b) lateral view of the plain radiograph showing Meyers type III tibial ACL avulsion fractures. (c) Coronal and (d) sagittal views of CT showing rounded bone fragments and osteosclerosis at the fracture site, suggesting that the fracture is old. ACL: anterior cruciate ligament; CT: computed tomography.

**Figure 2:**
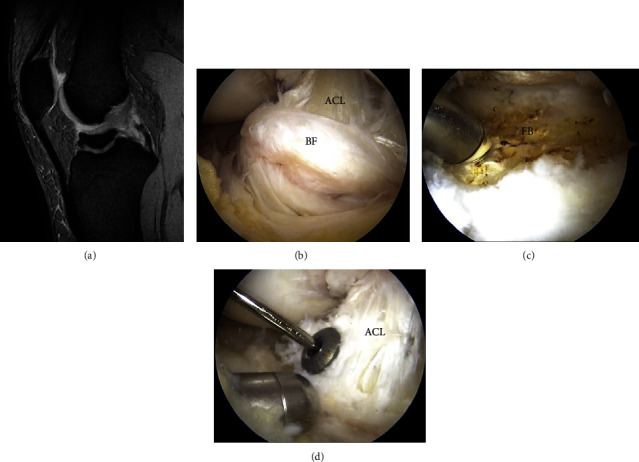
(a) Preoperative STIR image of MRI showing poor delineation of the ACL. (b) Arthroscopic image of the ununited fracture area. The bone fragment was displaced. (c) Arthroscopic image of the fracture bed. The fracture bed was freshened using a 4.5 mm arthroscopic shaver and a bipolar electrode. (d) Arthroscopic image of the fixation. The bone fragment was fixed via screw fixation. STIR: short T-inversion recovery; MRI: magnetic resonance imaging; ACL: anterior cruciate ligament; BF: bone fragment; FB: fracture bed.

**Figure 3:**
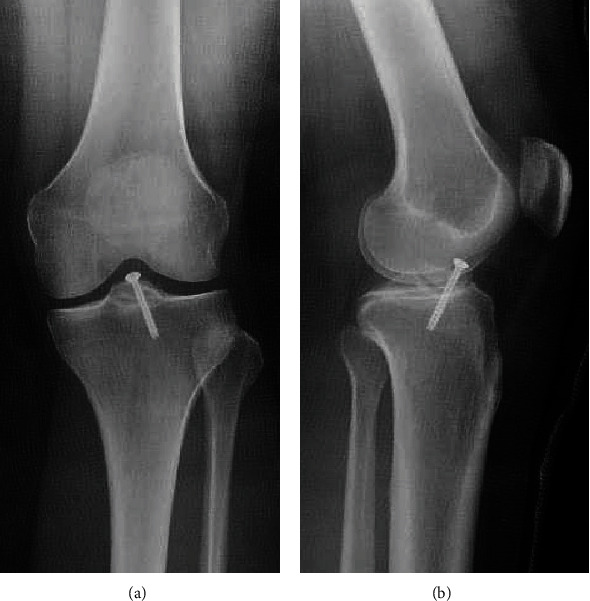
(a) Frontal view and (b) lateral view of plain radiography showing good repositioning was confirmed.

Imaging studies performed included radiography, computed tomography (CT), and magnetic resonance imaging (MRI). Radiography showed Meyers' type III tibial ACL avulsion fractures (Figures 1(a) and 1(b)) [8]. CT revealed rounded bone fragments and osteosclerosis at the fracture site, suggesting that it was an old fracture (Figures 1(c) and 1(d)). MRI revealed poor delineation of the ACL (Figure 2(a)). An osteosynthesis of the fracture nonunion was performed arthroscopically. The displacement of the bone fragments of the intercondylar eminence fracture was confirmed arthroscopically (Figure 2(b)). Anterior cruciate ligament (ACL) fibers were not ruptured. Both the displaced bone fragments and the fracture bed were sufficiently freshened using a 4.5 mm arthroscopic shaver (Merlin; Zimmer Biomet, Warsaw, IN, USA) and a bipolar electrode (Super MULTIVAC 50 3.75 mm; Smith and Nephew, Watford, HRT, England) (Figure 2(c)); the bone fragments were repositioned and fixed with a 4.0 mm cannulated cancellous screw (ACE Titanium Cannulated Cancellus Screw System; Zimmer Biomet, Warsaw, IN, USA) (Figure 2(d)). ACL degeneration was observed, but the tension was good. Postoperative radiography revealed good repositioning (Figure 3).

**Figure 4:**
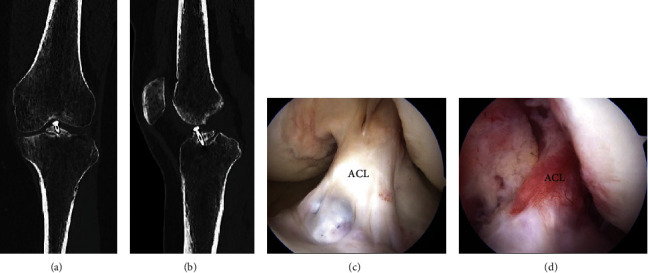
(a) Coronal and (b) sagittal views of CT at 4 months postoperatively showing fusion of most of the bones. MRI at 6 months postoperatively. (c) Arthroscopic image at screw removal showing that good ACL volume and tension were maintained. (d) Arthroscopic image (acquired after deflating the tourniquet) showing that blood flow was confirmed in the ACL. CT: computed tomography; MRI: magnetic resonance imaging; ACL: anterior cruciate ligament.

Postoperative cast immobilization in mild flexion (30 degrees) and nonweight-bearing was performed for 3 weeks. ROM exercises were then performed. A rigid frame brace with 10-degree extension limitation and no flexion limitation was applied, starting at 3 weeks postoperatively with 1/3 weight-bearing, followed by 1/2 weight-bearing at 5 weeks postoperatively, 2/3 weight-bearing at 6 weeks postoperatively, and full weight-bearing at 8 weeks postoperatively. At 4 months postoperatively, CT revealed union (Figures 4(a) and 4(b)). At 5 months postoperatively, the brace was ended. At 6 months postoperatively, she had no pain, instability had disappeared with the anterior drawer test, and she had a hard endpoint. Screw removal was performed via arthroscopy. Arthroscopically, good ACL volume and tension were maintained (Figure 4(c)). Furthermore, when the tourniquet was released, blood flow could be seen in the ACL (Figure 4(d)). The patient's condition was still good. The knee injury and osteoarthritis outcome score (KOOS) improved from 24 points preoperatively to 83 points postoperatively.

## 3. Discussion

Nonunited tibial ACL avulsion fractures are rare, and the optimal treatment for such fractures has not yet been well established [2]. In addition, to date, there have been no reports of the treatment of tibial ACL avulsion fracture nonunion 30 years after the injury. This is the first report of a tibial ACL avulsion fracture nonunion 30 years after the injury in which good results were obtained with screw fixation via arthroscopy.

Tibial ACL avulsion fractures cause knee pain, instability, and limited extension [4, 5]. They are classified into three types per the Meyers and McKeever classification. Type I indicates almost no displacement, type II indicates a raised anterior fragment but continuity posteriorly, and type III indicates complete rupture and displacement. Types I and II with continuity are indicated for conservative treatment, whereas type III with displacement is indicated for surgical treatment [8].

The treatment of tibial ACL avulsion fracture nonunion has been reported to include debridement with arthroscopy for limitation of extension, as well as osteosynthesis by open reduction, ACL reconstruction, and arthroscopic reduction and pull-out sutures for instability and pain [4–7]. Atusmi et al. reported that arthroscopic debridement of the anterior scar of a bone fragment improved extension limitation in a case of tibial ACL avulsion fracture nonunions with extension limitation as the primary complaint [6]. This has the advantage of being minimally invasive and is a good indication for middle-aged and older patients who are not highly active in sports [6]. Keys and Walters reported a case of nonunited tibial ACL avulsion fractures with a one-year-long pain on extension, which was successfully treated with fresh bone fragments, bone grafting, and screw fixation by open surgery [4]. Horibe et al. performed ACL reconstruction in three tibial ACL avulsion fracture nonunions for anterior instability and limited extension. In these cases, anterior instability and limitation of extension were improved. They suggested that for tibial ACL avulsion fracture with anterior instability, arthroscopic debridement/notchplasty alone is not sufficient, and ACL reconstruction is a better option [7]. Ahn and Yoo performed arthroscopic reduction and pull-out suture on 14 fresh and nonunited tibial ACL avulsion fractures and improved extension limitation and anterior instability in all cases [5]. The pull-out suture can be performed regardless of the size of the bone fragments, even in cases where the fragments are crushed [5]. We confirmed that the ACL was not ruptured under arthroscopy, after which we refreshed the fracture site to promote bony healing, followed by screw fixation. Good results were obtained with screw fixation.

Nonunion is defined by the Food and Drug Administration as “a fracture that shows no signs of healing for at least 3 months and persists for more than 9 months,” and this definition is widely used [9]. Nonunion can be caused by biological factors, the fracture type, and inadequate reduction and fixation [10]. Nonunion may occur in joints due to joint fluid or synovial membrane. Nonunion caused by fluid-filled, membrane-covered cavities formed between fracture fragments is known as synovial pseudarthrosis [11]. Debridement, bone grafting, and internal fixation are the most common surgical treatment options for nonunion [12]. Our success in obtaining bony fusion of a tibial ACL avulsion fracture nonunion can be attributed to adequate debridement, reduction, and internal fixation.

This is the first report of a tibial ACL avulsion fracture nonunion that had been sustained for 30 years after the initial injury and was successfully treated using a screw-fixation technique via arthroscopy. Appropriate debridement, reduction, and internal fixation resulted in union.

## Data Availability

The data presented in this study are available on reasonable request from the corresponding author. The data are not publicly available because they contain information that could compromise the privacy of the patient.
